# Regional myocardial function at preclinical disease stage of hypertrophic cardiomyopathy in female gene variant carriers

**DOI:** 10.1007/s10554-020-02156-1

**Published:** 2021-02-09

**Authors:** Rahana Y. Parbhudayal, Celine Seegers, Pierre Croisille, Patrick Clarysse, Albert C. van Rossum, Tjeerd Germans, Jolanda van der Velden

**Affiliations:** 1 Department of Cardiology, Amsterdam UMC, Amsterdam Cardiovascular Sciences, Vrije Universiteit Amsterdam, Amsterdam, The Netherlands; 2 Department of Physiology, Amsterdam UMC, Amsterdam Cardiovascular Sciences, Vrije Universiteit Amsterdam, De Boelelaan 1117, 1081 HV Amsterdam, The Netherlands; 3 The Netherlands Heart Institute, Utrecht, The Netherlands; 4 Univ Lyon, UJM‐Saint‐Etienne, INSA, CNRS UMR 5520, INSERM U1206, CREATIS, 42023 Saint‐Etienne, France

**Keywords:** Hypertrophic cardiomyopathy, Tissue tagging, *MYBPC3*, *MYH7*, *TNNT2*

## Abstract

We recently showed more severe diastolic dysfunction at the time of myectomy in female compared to male patients with obstructive hypertrophic cardiomyopathy. Early recognition of aberrant cardiac contracility using cardiovascular magnetic resonance (CMR) imaging may identify women at risk of cardiac dysfunction. To define myocardial function at an early disease stage, we studied regional cardiac function using CMR imaging with tissue tagging in asymptomatic female gene variant carriers. CMR imaging with tissue tagging was done in 13 *MYBPC3*, 11 *MYH7* and 6 *TNNT2* gene carriers and 16 age-matched controls. Regional peak circumferential strain was derived from tissue tagging images of the basal and midventricular segments of the septum and lateral wall. Left ventricular wall thickness and global function were comparable between *MYBPC3*, *MYH7*, *TNNT2* carriers and controls. *MYH7* gene variant carriers showed a different strain pattern as compared to the other groups, with higher septal peak circumferential strain at the basal segments compared to the lateral wall, whereas *MYBPC3*, *TNNT2* carriers and controls showed higher strain at the lateral wall compared to the septum. Only subtle gene-specific changes in strain pattern occur in the myocardium preceding development of cardiac hypertrophy. Overall, our study shows that there are no major contractile deficits in asymptomatic females carrying a pathogenic gene variant, which would justify the use of CMR imaging for earlier diagnosis.

**Supplementary Information:**

The online version of this article (10.1007/s10554-020-02156-1) contains supplementary material, which is available to authorized users.

## Introduction

Hypertrophic cardiomyopathy (HCM) is the most common genetic cardiomyopathy with an autosomal dominant pattern of inheritance [1]. HCM typically presents with asymmetric left ventricular hypertrophy (LVH) most frequently at the basal septum, in the absence of any abnormal loading conditions [2]. A causative gene variant (i.e. mutation) in genes encoding sarcomere proteins is identified in approximately 50–60% of all index patients (genotype-positive individuals) [3]. In the majority of patients, variants in the genes encoding thick filament proteins myosin binding protein-C (*MYBPC3*) and β-myosin heavy chain (*MYH7*) and the thin filament protein troponin T (*TNNT2*) are found [4]. We recently observed more severe diastolic dysfunction in female compared to male patients with obstructive hypertrophic cardiomyopathy at the time of cardiac surgery [5]. Correction of cardiac dimensions by body surface area (BSA) revealed more severe cardiac remodeling in female compared to male patients evident from a significantly higher BSA-indexed left atrial dimension and BSA-indexed septal thickness. A subsequent study in a cohort of genotype-positive subjects referred for family screening indicated that correcting maximal wall thickness for body size and applying specific cut-off values improved the predictive accuracy for HCM-related events [6]. These recent studies indicate that females may be underrepresented in HCM patient studies because of the current HCM diagnostic criterium of ≥ 15 mm LV wall thickness (≥ 13 mm in case of first-degree family members) [2], which does not take into account body size [7]. Indeed, the percentage of female patients in HCM patient cohort studies is on average 30–40% [8–10], which may be explained by lower disease penetrance, but could also imply that cardiac dysfunction remains undetected, in particular in the female HCM patient group, using cardiac remodeling, i.e. hypertrophy, rather than cardiac dysfunction as diagnostic criterium.

To detect early gene variant-related functional changes in in vivo cardiac function, studies are warranted in asymptomatic gene variant carriers without cardiac remodeling (i.e. no hypertrophy, no fibrosis, no capillary rarefaction) using advanced cardiac imaging. Here, we used cardiovascular magnetic resonance (CMR) imaging with high resolution tissue tagging to investigate if regional myocardial functional differences exist in female asymptomatic gene variant carriers who harbor common HCM gene variants in thick (*MYBPC3*, *MYH7*) and thin (*TNNT2*) filament genes.

## Material and methods

The CMR imaging studies in this study included 30 female asymptomatic carriers with gene variants in *MYBPC3* (n = 13), *MYH7* (n = 11) and *TNNT2* (n = 6). Gene variant carriers were included after genetic screening, classified as likely pathogenic and pathogenic (clinically graded class 4 or 5), and were first-degree relatives of index HCM patients. All gene variant carriers had a wall thickness of the LV < 13 mm (based on ESC guidelines) and were free of any systemic and/or cardiac disease and used no medication. Data from gene variant carriers were compared with data from 16 healthy female controls, who were age and gender matched. Out of the 46 study participants 4 controls, 2 *MYBPC3* gene variant carriers, 4 *MYH7* gene variant carriers and 2 *TNNT2* gene variant carriers were interrelated. All participants underwent a CMR imaging protocol. The study conformed to the principles outlined in the Declaration of Helsinki and was approved by the Medical Ethical Committee of the VU University Medical Center Amsterdam. Written informed consent was obtained from all study participants. The STROBE checklist has been used for preparing the manuscript.

### Cardiovascular magnetic resonance imaging

CMR imaging was performed using a 1.5 T whole body scanner (Avanto, Siemens, Erlangen, Germany), with a six-channel phased-array body coil. A stack of short axis cines was used for LV full coverage. Cine images were acquired in a single breath-hold using a balanced segmented steady-state free precession (SSFP) [11]. Also, 4, 3 and 2 chamber long axis SSFP cine images were obtained. From the short axis cine images LV end-diastolic and end-systolic volumes and mass were obtained. Typical image parameters were: 5 mm slice thickness with 5 mm gap between short-axis slices, temporal resolution < 50 ms, repetition time 3.2 ms, echo time 1.54 ms, flip angle 60°, and typical image resolution 1.3 × 1.6 mm.

**Fig. 1:**
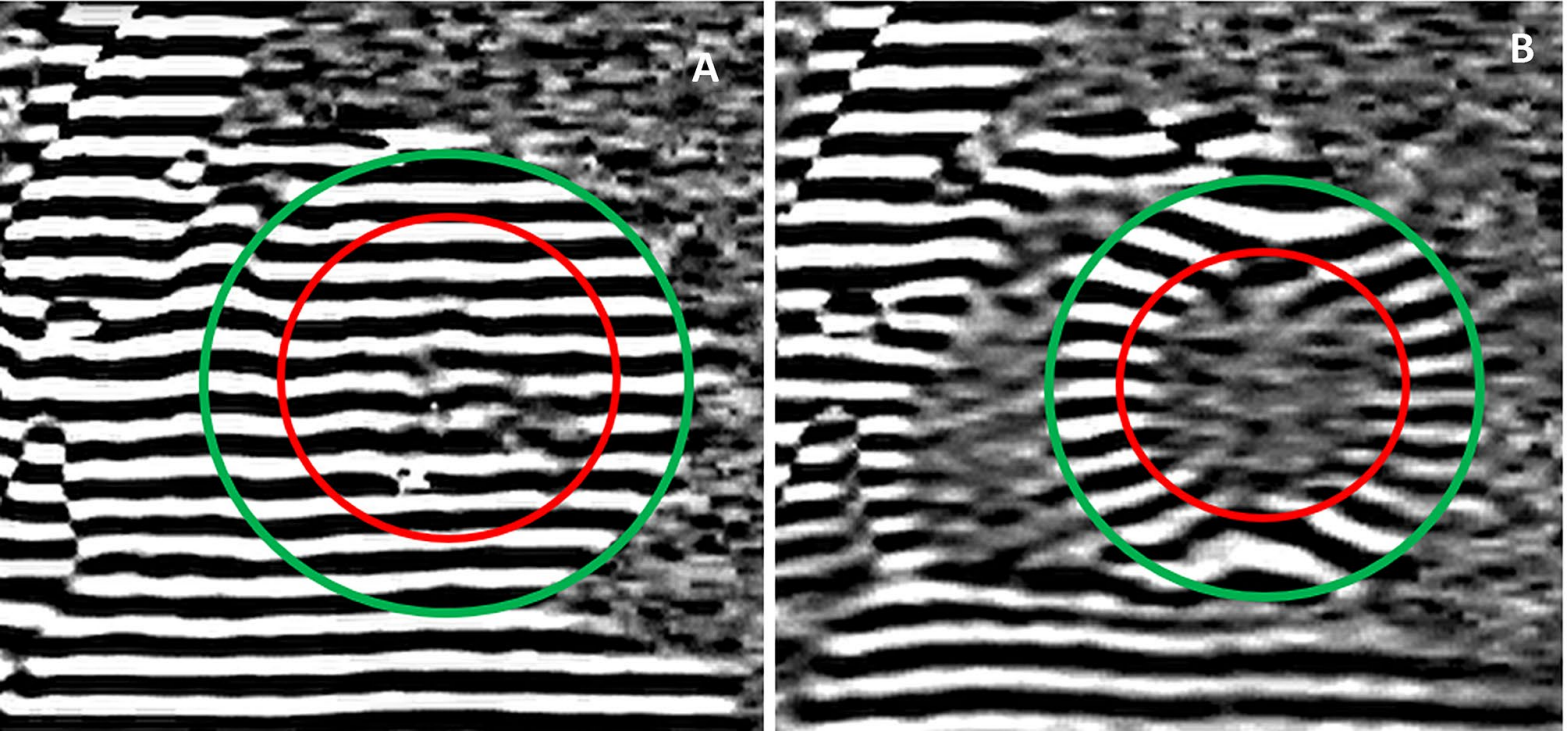
SSFP Myocardial tissue tagging. **a** At end-diastole, a line tagging grid is applied. The myocardium is delineated by the epicardial (green circle) and endocardial (red circle) contours. **b** As the taglines are a temporary property of the myocardium, deformation (strain) can be depicted and quantified by this method, as illustrated by this end-systolic image

**Fig. 2:**
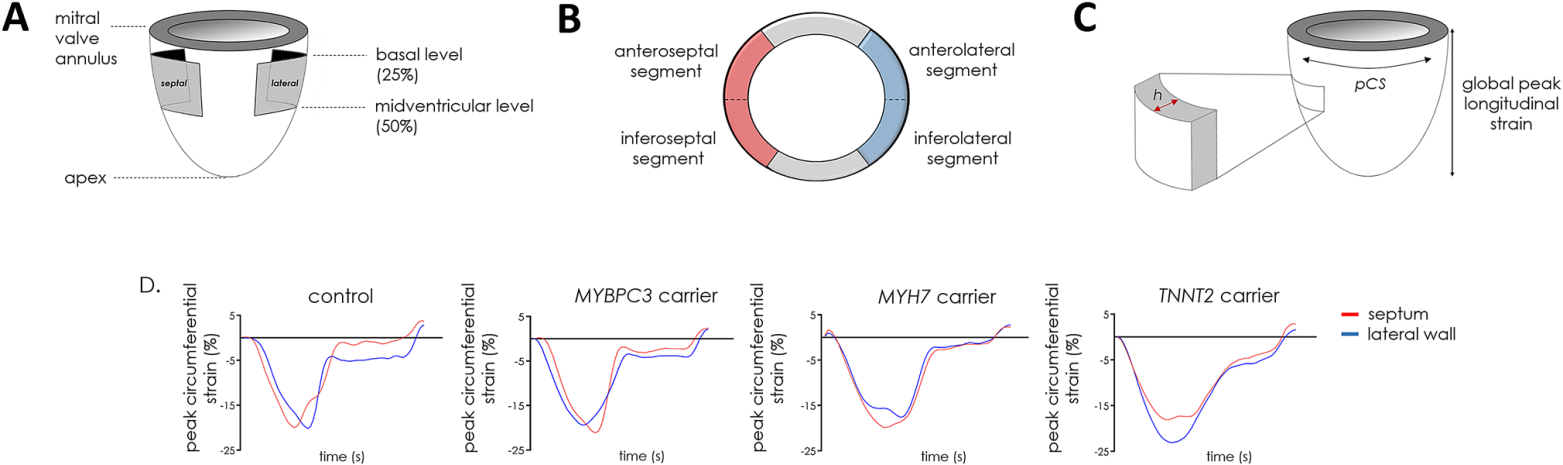
Long and short axis images of the left ventricle. **a** Schematic image of a long axis of the left ventricle. Depicted are the two positions of the basal (25%) and midventricular (50%) levels where myocardial tissue tagging was applied. **b** Four septal segments (two anteroseptal and two inferoseptal) were compared to the four lateral segments (two anterolateral and two inferolateral) at basal and midventricular level **c** Schematic image of global peak longitudinal strain. At basal and midventricular level, end-diastolic wall thickness (*h*) and peak circumferential strain (pCS) were measured according to the 17 segment AHA classification. **d** Representative peak circumferential strain curves of one healthy control subject, one *MYBPC3*, one *MYH7* and one *TNNT2* gene variant carrier are shown. The red curves indicate the septum. The blue curves indicate the lateral wall

For regional function assessment, myocardial tissue tagging imaging was performed using a multiple breath-hold, retrospectively triggered SSFP myocardial tissue tagging sequence with the linearly increasing start-up approach [12]. See Fig. 1. Two short axis planes were positioned at 25 and 50 percent of the distance between the mitral valve annulus and the apex (Fig. 2a). Image parameters were: 7 mm slice thickness, temporal resolution 14 ms, repetition time 4.7 ms, echo time 2.3 ms, flip angle 20°, and in-plane image resolution of 1.2 by 3.8 mm, with 7 mm tag spacing.

Late Gadolinium enhancement images were obtained 10 min after injection of 0.2 mmol/kg Gadolinium-DTPA. An inversion recovery Fast low angle shot sequence was used to obtain images with 6 mm slice thickness planned in the same orientation as the long and short axis cines.

### Post processing

LV volumes and mass analysis were performed by a single investigator, using Circle CVi42, Calgary, Canada. Endocardial contours were drawn to calculate LV end-diastolic (LVEDV) and end-systolic volumes (LVESV) and ejection fraction (LVEF). Epicardial contours were added to calculate LV end-diastolic wall thickness and LV mass. Papillary muscles were included in LV volumes and excluded from LV mass. LV end-diastolic, end-systolic volumes and LV mass were indexed for body surface area. End-diastolic wall thickness at the septum and lateral wall were derived from respectively the mean of four septal (anteroseptal and inferoseptal) and lateral segments (anterolateral and inferolateral) at the basal and midventricular level.

Circumferential strain analysis was obtained from the 50% mid myocardial layer from tissue tagging cines (Fig. 2b), using Intag software (CREATIS, Lyon, France), and has been reported to be most reproducible [13, 14]. LV segmentation was performed according to the 17 segment AHA model [15]. From this analysis, peak circumferential strain and peak diastolic circumferential strain rate per segment were obtained. The four septal segments (at basal and midventricular level antero- and inferoseptal segments) were compared with the four lateral segments (at basal and midventricular level antero- and inferolateral segments) (Fig. 2b). Representative peak circumferential strain curves of 1 healthy control subject and 3 gene variant carriers are shown in Fig. 2d (red curves indicate the septum and blue curves indicate the lateral wall). Global longitudinal strain was obtained from the 4, 3 and 2 chamber long axis cines with tissue tracking using CVi 42 software (Circle Cardiovascular Imaging, Calgary, Canada) (Fig. 2c).

### Statistical analysis

Statistical analysis was performed using SPSS software (version 22.0; SPSS, Chicago, IL, USA). Normality of data was inspected visually by means of QQ-plots. Means of continuous demographic and outcome variables were compared between gene variant carrier groups using ANOVA with a Bonferroni post-hoc analysis after normality was verified. Exact chi-square test was used for categorical demographic variables. A mixed model analyses was used to test whether regional differences in mean wall thickness and peak circumferential strain differed between gene variant carriers groups and controls. The model included fixed effects for gene variant group, region (septal or lateral) and their two-way interaction and a random effect for subject. In case of a significant two-way interaction, post-hoc analysis with Bonferroni correction were performed to test for regional differences within each gene variant carrier and control group separately. As two separate statistical tests were performed for basal and midventricular segments a two-sided significance level of 0.05/2 was used for all statistical tests to account for multiple testing. For baseline characteristics a significance level of < 0.05 was used.

## Results

Table 1 summarizes genetic and clinical parameters of all study participants. Overall, controls and carriers were of similar age and had similar BSA. No differences were present in cardiac function (ejection fraction, stroke volume) and left ventricular mass between controls and carrier groups. LVEDV and LVESV in *TNNT2* group were significantly smaller than in *MYBPC3*, but similar to controls and *MYH7* (Table 1). None of the gene variant carriers and controls showed contrast enhanced myocardial areas.

**Table 1:** Demographics and left ventricular parameters

	Controls (n = 16)	*MYBPC3* carriers (n = 13)	*MYH7* carriers (n = 11)	*TNNT2* carriers (n = 6)
Genotype	No genotype	c.2373dupG (*n* = 13)	c.4130C > T (*n* = 5)	c.304C > T (*n* = 3)
			c.5135G > A (*n* = 2)	c.856C > T (*n* = 1)
			c.1207C > T (*n* = 3)	c.403C > T (*n* = 1)
			c.1727A > G (*n* = 1)	c.277G > A (*n* = 1)
Age	44 ± 12	37 ± 14	38 ± 14	43 ± 15
BSA (m^2^)	1.80 ± 0.09	1.73 ± 0.11	1.78 ± 0.17	1.77 ± 0.19
LVEDV (ml·m^−2^)	75.8 ± 10.2	83.6 ± 7.3	79.6 ± 8.9	69.6 ± 15.5*
LVESV (ml·m^−2^)	25.1 ± 4.9	28.4 ± 5.8	26.2 ± 4.4	19.8 ± 5.1*
SV (ml·m^−2^)	50 ± 8	55 ± 9	53 ± 8	50 ± 11
LV mass (g·m^−2^)	36.8 ± 6.5	55.1 ± 9.3	53.3 ± 7.6	49.8 ± 11.3

Data are presented as mean ± standard deviation. *MYBPC3* myosin binding protein C gene, *MYH7* myosin heavy chain gene, *TNNT2* troponin T gene. *BSA* body surface area, *LVEF* left ventricular ejection fraction, *SV* stroke volume. *p < 0.05 versus *MYBPC3*

### Regional anatomical parameters

End-diastolic wall thickness of basal and midventricular segments of the septum and lateral wall were comparable between *MYBPC3*, *MYH7* and *TNNT2* groups and controls (Table 2). In addition, septal-to-lateral wall thickness (S/L) ratio of basal and midventricular segments of the septum and lateral wall were comparable between gene variant carrier groups and controls (Table 2).

**Table 2:** Regional anatomical differences between gene variant carriers and controls

	Controls (n = 16)	*MYBPC3* carriers (n = 13)	*MYH7* carriers (n = 11)	*TNNT2* carriers (n = 6)
Basal level				
Septum				
EDWT (mm)	5.9 ± 0.5	5.7 ± 1.0	5.2 ± 0.9	5.2 ± 1.3
EDWT (mm·m^−2^)	3.3 ± 0.4	3.3 ± 0.5	2.9 ± 0.5	2.9 ± 0.4
Lateral wall				
EDWT (mm)	5.6 ± 0.7	5.3 ± 0.7	5.4 ± 0.7	5.5 ± 1.2
EDWT (mm·m^−2^)	3.1 ± 0.4	3.1 ± 0.4	3.0 ± 0.3	3.1 ± 0.6
S/L ratio	1.17 ± 0.1	1.1 ± 0.2	1.0 ± 0.1	1.0 ± 0.3
Midventricular level				
Septum				
EDWT wall (mm)	5.4 ± 0.7	5.1 ± 0.9	5.3 ± 1.0	5.5 ± 1.3
EDWT (mm·m^−2^)	3.3 ± 0.4	3.3 ± 0.5	2.9 ± 0.5	2.9 ± 0.4
Lateral wall				
EDWT (mm)	4.6 ± 0.6	4.2 ± 0.5	4.3 ± 0.6	4.1 ± 0.6
EDWT (mm·m^−2^)	3.1 ± 0.4	3.1 ± 0.4	3.0 ± 0.3	3.1 ± 0.6
S/L ratio	1.2 ± 0.1	1.2 ± 0.2	1.2 ± 0.2	1.2 ± 0.1

Data are presented as mean ± standard deviation. EDWT: end-diastolic wall thickness; S/L: septum-to-lateral wall thickness. *MYBPC3*: myosin binding protein C gene; *MYH7*: myosin heavy chain gene; *TNNT2*: troponin T gene. None of the comparisons reached significance

### Regional functional parameters

**Fig. 3:**
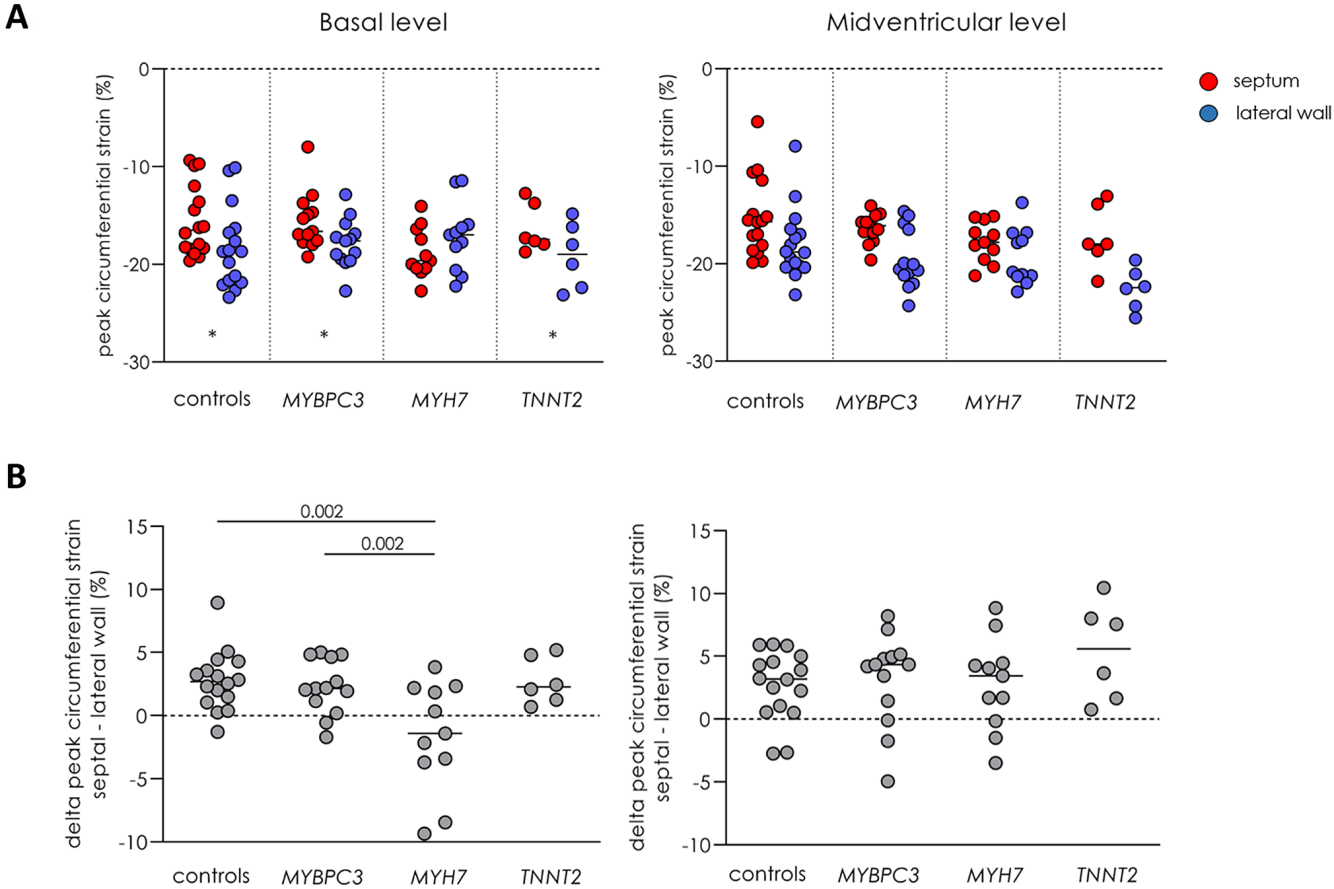
Regional functional differences between gene variant carriers and controls. **a** The mean of peak circumferential strain of two septal or lateral segments at the basal and midventricular level, and **b** the mean of the difference in peak circumferential strain between septum and lateral wall at basal and midventricular level. Data are presented as mean with standard deviation.*p < 0.025 septum vs. lateral wall

Global longitudinal strain was similar between *MYBPC3*, *MYH7* and *TNNT2* gene variant carriers (− 21.5 ± 2.2, − 23.0 ± 1.9 and − 22.0 ± 3.1%, respectively) and controls (− 21.4 ± 2.0%). Analysis of peak circumferential strain showed higher strain for the lateral segments compared to septal segments, both at basal and midventricular level, with significant differences between the septum and lateral segments at basal level in the *MYBPC3*, *TNNT2* gene variant carriers and controls (Fig. 2a, Table S1). Overall, this regional (septum vs. lateral wall) difference was observed in all gene variant carrier groups and controls, except for the *MYH7* gene variant group at basal level which showed an opposite pattern with a higher strain in the septum than in the lateral segments (Fig. 3a, Table S1). The difference in peak circumferential strain between septal and lateral segments was calculated per individual and is depicted in Fig. 3b. The delta (difference between septum and lateral wall) is similar in all groups, except for the *MYH7* gene variant group at basal level. The value in the *MYH7* gene variant group is significantly different from the value observed in the *MYBPC3* group and controls (Fig. 3b). Peak diastolic circumferential strain rate of the basal and midventricular segments of the septum and lateral wall were comparable between gene variant carrier groups and controls (Table S2).

## Discussion

Our case–control study in preclinical female variant carriers using state-of-the-art cardiac imaging shows a subtle change in cardiac function only in individuals with a *MYH7* gene variant. Previous studies reported myocardial alterations in asymptomatic gene variant carriers, although these studies did not specify genotype or sex (summarized in Table 3) [16–31]. These alterations include differences in anatomical and functional level, such as as the amount fibrotic tissue or number of clefts in the myocardium and different length of the anterior mitral valve leaflet [16, 20–28], and a higher LV ejection fraction and torsion and altered myocardial metabolism [17–19, 29–31].

**Table 3:** Anatomic and functional changes reported in gene variant carriers

Genes	Number of gene variant carriers (male/female)		Reference
*Anatomic changes*			
Not defined	15 (10/5)	Gene variant carriers showed a limited amount of fibrosis on CMR imaging compared with overt HCM and therefore seemed closely linked to disease development in HCM	Moon et al. [20]
*MYBPC3* and *TPM1*	16 (not defined)	Profound crypts in the inferoseptum in gene variant carriers	Germans et al. [21]
*MYBPC3* and *TPM1*	43 (not defined)	Crypts in gene variant carriers showed deeper penetrance than controls	Brouwer et al. [22]
Not defined	20 (not defined)	No structural abnormalities described (crypts) In gene variant carriers compared with controls	Petryka et al. [23]
*MYBPC3* (15), *MYH7* (7), *TNNT2* (3), *MYL2* (2), *TPM1* (1), multiple genes (2)	30 (9/21)	Prevalence of an accessory LV apical-basal muscle bundle in gene variant carriers was significantly higher than in control subjects	Gruner et al. [24]
*MYBPC3* (18), *MYH7* (7), *TNNT2* (4), *TNNI3* (7), *ACTC1* (2), multiple genes (1)	39 (15/24)	Increased apical LV trabecular complexity, higher amount of myocardial clefts and a higher LV ejection fraction in gene variant carriers compared to healthy subjects	Captur et al. [25]
*MYBPC3* (31), *MYH7* (23), *TNNT2* (7), *TNNI3* (9), *ACTC1* (3), *MYL3* (1)	73 (37/36/ 1 unknown)	The combined presence of ≥ 2 myocardial crypts, ≥ 21 mm anterior mitral valve leaflet length, increased LV apical trabecular complexity and smaller LV end-systolic volume is indicative of gene variant carriers. *MYBPC3* gene variant carriers have a twofold prevalence of crypts and less LV systolic cavity reduction compared to the other gene variant carriers with other than *MYBPC3* gene variants	Captur et al. [16]
*MYBPC3* (16), M*YH7* (6), *TNNT2* (4), *TNNI3* (7), *ACTC1* (2), multiple genes (1)	36 (12/24)	Although within normal values, septal wall thickness was higher in gene variant carriers compared to controls. Additionally, gene variant carriers were reported to have a higher amount of myocardial crypts, increase in septal convexity, longer anterior mitral valve leaflet and a smaller LV end-systolic volume than healthy controls. Comparisons between thick and thin filament affected gene variant carriers revealed a greater septal convexity in thick filament gene variant carriers	Reant et al. [26]
*MYBPC3*	47 (3/44)	No differences in length of posterior mitral valve leaflet were detected compared to healthy controls	Tarkiainen et al. [27]
*MYBPC3* (13), *MYH7* (12), *TNNT2* (2), *TNNI* (1)	28 (3/25)	Extracellular volume was higher in gene variant carriers compared with healthy controls	Hiremath et al. [28]
*Functional changes*			
*MYBPC3* (15), *MYH7* (19),*TNNT2* (5)	39 (16/23)	Gene variant carriers revealed increased myocardial collagen synthesis evident from elevated levels of serum pro-peptide of type I collage (PICP) compared with controls. This increase was significantly higher in *MYH7* than *MYBPC3* gene variant carriers which agreed with a larger reduction in diastolic dysfunction in *MYH7* than *MYBPC3* gene variant carriers	Ho et al. [17]
*MYBPC3* (22), *TPM1* (6)	28 (11/17)	Septal to lateral-ratio was larger in gene variant carriers compared to controls. Contractility was higher in basal inferolateral segments than in controls. While controls revealed a significant difference in contractile function between septal and lateral, this was blunted in gene variant carriers. Gene variant carriers showed lower diastolic function compared with controls, pronounced at basal slice of the LV	Germans et al. [29]
*MYBPC3* (13), *TPM1* (4)	17 (5/12)	Increased LV ejection fraction, torsion and the ratio of peak LV torsion to peak endocardial circumferential shortening (TECS-ratio) in gene variant carriers than healthy controls	Russel et al. [30]
*MYBPC3*	15	As in healthy controls, *MYBPC3* gene variant carriers also revealed a heterogeneous contraction pattern between anterior and lateral region. There were no differences in contractile function between *MYBPC3* gene variant carriers and controls. Gene variant carriers revealed impaired myocardial energetics compared with controls	Timmer et al. [18]
*MYBPC3* (14), *MYH7* (12), *TNNT2* (3)	29 (17/12)	Extracellular volume (ECV) was increased in the absence of focal fibrosis detected on CMR imaging in gene variant carriers compared to controls. There were no differences observed in ECV between *MYBPC3* and *MYH7* gene variant carriers	Ho et al. [31]
*MYBPC3* (14), *MYH7* (14)	28 (7/19)	*MYH7* gene variant carriers revealed lower external work and myocardial external efficiency (MEE) than *MYBPC3* gene variant carriers. MEE was lower in gene variant carriers compared to healthy controls	Witjas-Paalberends et al. [19]

Strain measurements in asymptomatic carriers harboring thick filament gene variants, demonstrated comparable global and regional systolic strain as observerd in healthy controls [32]. A sub-analysis in the latter study comparing 35 *MYH7* with 24 *MYBPC3* gene variant carriers revealed a younger study population and higher peak longitudinal strain in *MYH7* compared to *MYBPC3* gene variant carriers [32]. While Ho et al. have not specified the mechanism in the higher global longitudinal stain in *MYH7* gene carriers [32], it may be speculated that a higher systolic strain at a regional level may explain this observation.

A previous study from our group investigating the effect of thick and thin filament gene variants (*MYH7* and *MYBPC3*) associated with HCM on human cardiac myofilament function, demonstrated significantly higher tension cost, i.e. the amount of energy used during force development, in *MYH7* compared to *MYBPC3* [19], which coincided with a larger reduction in in vivo myocardial external efficiency compared to the control group in *MYH7* than in *MYBPC3* carriers. Follow-up studies showed that the reduction in myocardial external efficiency is present in individuals with thick and thin filament gene variants, and is explained by an increased cardiac oxygen consumption rather than altered contractile properties [33, 34]. The present study shows a subtle change in the contraction pattern in preclinical female *MYH7* carriers, which was not seen in *MYBPC3* carriers. While this subtle change in *MYH7* carriers may in part explain the gene-specific difference in cardiac efficiency [19], the current and previous studies [20, 34] indicate that changes in energy consumption (i.e. increased oxygen consumption) rather than perturbations in (regional) contractile properties of the heart muscle characterize the very early disease stage of HCM.

With respect to gene variant-specific in vitro findings, functional properties of sarcomeres affected by thick-filament gene variants, obtained from tissue of HCM patients who underwent septal myectomy, revealed lower maximal force production in cardiac muscle strips containing *MYH7* gene variants than in tissue with *MYBPC3* gene variants [19]. Additionally, compared to tissue from genotype mutation-negative HCM patients, *MYH7* affected sarcomere gene variants revealed increased kinetics of tension development [35, 36]. Also, as HCM is most frequently inherited through a heterogeneous manner, allelic transcription, which occurs in a stochastic manner, may lead to variable expression of healthy and mutant proteins [37, 38] and may cause inhomogeneous contraction and relaxation. On in vivo cardiac imaging, this may lead to an increase in regional circumferential strain as seen in our study cohort and longitudinal strain observed in previous work [32]. However, the step from in vitro sarcomere function to in vivo circumferential strain imaging may be too large, since the effect of extracellular volume in the myocardium and myofiber disarray are challenging to take into account in in vitro experiments.

### Limitations

The number of recruited carriers were limited, therefore, very subtle functional differences may have remained undetected. However, myocardial tissue tagging is a robust and sensitive method to evaluate regional function, and therefore the clinical value of subtle differences not detected with the method with these number of carriers is limited. In addition, controls were not genotyped. There is a small chance that within this group op controls, unidentified carriers were present.

## Conclusions

Overall, CMR combined with tissue tagging detects subtle gene-specific regional differences in contractility. However, assessment of regional contraction by CMR tissue tagging currently does not aid in the identification of early cardiac disease changes in the preclinical genotype-positive population. Moreover, our study shows that there are no major contractile deficits in asymptomatic females carrying a pathogenic gene variant, which would justify the use of CMR imaging for earlier diagnosis.

## Supplementary Information

Below is the link to the electronic supplementary material.

Electronic supplementary material 1 (DOCX 35 kb)

## Abbreviations

CMR: Cardiovascular magnetic resonance
HCM: Hypertrophic cardiomyopathy
LVEF: Left ventricular ejection fraction
LVH: Left ventricular hypertrophy
*MYBPC3*: Myosin binding protein-C gene
*MYH7*: β-Myosin heavy chain gene
*TNNT2*: Troponin T gene
