# Supplementary material for: Regional myocardial function at preclinical disease stage of hypertrophic cardiomyopathy in female gene variant carriers
Source: Int J Cardiovasc Imaging. 2021 Feb 9;37(6):2001–10. doi: 10.1007/s10554-020-02156-1 (PMC8255263; doi:10.1007/s10554-020-02156-1)
Supplement: Supplementary file 1 — Electronic supplementary material 1 (DOCX 35 kb) [file 10554_2020_2156_MOESM_ESM.docx]

**Regional myocardial function at preclinical disease stage of hypertrophic cardiomyopathy in female gene variant carriers**

R.Y. Parbhudayal, MD^a,b,c^, C. Seegers, BSc^a^, P. Croisille, MD, PhD^d^, P. Clarysse, PhD^d^, A.C. van Rossum, MD, PhD^a,c^, T. Germans, MD, PhD^a^, J. van der Velden, PhD^b,c^

^a^Department of Cardiology, ^b^Department of Physiology, Amsterdam UMC, Vrije Universiteit Amsterdam, Amsterdam Cardiovascular Sciences, The Netherlands

^c^The Netherlands Heart Institute, Utrecht, The Netherlands

^d^ Univ Lyon, UJM‐Saint‐Etienne, INSA, CNRS UMR 5520, INSERM U1206, CREATIS, F‐42023, Saint‐Etienne, France

**SUPPLEMENTAL MATERIAL**

**Table S1. Regional peak circumferential strain**

|  | | **Controls (n=16)** | ***MYBPC3* carriers (n=13)** | ***MYH7* carriers (n=11)** | ***TNNT2* carriers (n=6)** |
| --- | --- | --- | --- | --- | --- |
| **Basal segments** |  |  |  |  |  |
| *Septum* | Peak circumferential strain (%) | -15.6 ± 3.6 | -15.6 ± 2.9 | -18.8 ± 2.6 | -16.4 ± 2.5 |
| *Lateral wall* | Peak circumferential strain (%) | -18.3 ± 4.1 | -17.9 ± 2.5 | -17.2 ± 3.5 | -19.1 ± 3.3 |
| **Midventricular segments** | |  |  |  |  |
| *Septum* | Peak circumferential strain (%) | -15.3 ± 4.0 | -16.4 ± 1.5 | -17.8 ± 2.1 | -17.2 ± 3.2 |
| *Lateral wall* | Peak circumferential strain (%) | -18.0 3.6 | -19.6 ± 3.0 | -19.3 ± 2.9 | -22.6 ± 2.1 |

Data are presented as mean ± standard deviation. *MYBPC3*: myosin binding protein C gene; *MYH7*: myosin heavy chain gene; *TNNT2*: troponin T gene. BSA: body surface area; LVEF: left ventricular ejection fraction; SV: stroke volume. None of the comparisons reached significance.

**Table S2. Peak diastolic circumferential strain rate**

|  | | **Controls (n=16)** | ***MYBPC3* carriers (n=13)** | ***MYH7* carriers (n=11)** | ***TNNT2* carriers (n=6)** |
| --- | --- | --- | --- | --- | --- |
| **Basal segments** |  |  |  |  |  |
| *Septum* | Peak diastolic circumferential strain rate (%·s^-1^) | 35.1 ± 12.2 | 36.2 ± 9.6 | 36.5 ± 6.8 | 28.1 ± 6.9 |
| *Lateral wall* | Peak diastolic circumferential strain rate (%·s^-1^) | 40.5 ± 9.9 | 41.9 ± 10.8 | 37.7 ± 9.5 | 30.8 ± 6.5 |
| **Midventricular segments** | |  |  |  |  |
| *Septum* | Peak diastolic circumferential strain rate (%·s^-1^) | 36.4 ± 15.0 | 42.5 ± 12.7 | 37.5 ± 9.8 | 33.7 ± 8.6 |
| *Lateral wall* | Peak diastolic circumferential strain rate (%·s^-1^) | 37.7 ± 9.5 | 41.9 ± 10.8 | 39.2 ± 7.6 | 38.5 ± 5.9 |

Data are presented as mean ± standard deviation. *MYBPC3*: myosin binding protein C gene; *MYH7*: myosin heavy chain gene; *TNNT2*: troponin T gene. BSA: body surface area; LVEF: left ventricular ejection fraction; SV: stroke volume. None of the comparisons reached significance.

| Pat_ID | age | BSA | EDVi | ESVi | Svi | LVmassi | sept_pCS bas slice | lat_pCS basal_slice | sept_pCS mid_slice | lat_pCS mid_slice | sep_bas_WT | lat_bas_WT | sep__mid_WT | lat_mid_WT | SL rat bas | SL rat mid | GLS |
| --- | --- | --- | --- | --- | --- | --- | --- | --- | --- | --- | --- | --- | --- | --- | --- | --- | --- |
| control | 30 | 1,77 | 80,54 | 25,69 | 54,84 | 34,58 | -14,45 | -23,40 | -19,90 | -20,40 | 6,10 | 6,07 | 4,71 | 4,13 | 1,00 | 1,14 | -21,88 |
| control | 23 | 1,82 | 65,90 | 31,28 | 34,29 | 27,03 | -16,80 | -21,87 | -15,53 | -18,76 | 5,65 | 4,62 | 4,68 | 3,70 | 1,22 | 1,27 | -21,58 |
| control | 33 | 1,95 | 85,03 | 23,60 | 61,43 | 39,09 | -9,35 | -10,41 | -15,75 | -13,10 | 5,67 | 5,16 | 4,90 | 4,64 | 1,10 | 1,06 | -19,97 |
| control | 46 | 1,71 | 75,92 | 22,81 | 53,10 | 39,35 | -9,71 | -10,11 | -18,65 | -23,20 | 5,89 | 5,05 | 4,80 | 4,80 | 1,17 | 1,00 | -20,37 |
| control | 58 | 1,78 | 73,06 | 28,90 | 44,16 | 32,78 | -18,34 | -19,81 | -11,45 | -17,35 | 6,02 | 4,70 | 5,10 | 4,09 | 1,28 | 1,25 | -21,42 |
| control | 58 | 1,77 | 63,92 | 27,07 | 36,86 | 30,34 | -16,13 | -18,68 | -15,20 | -21,15 | 5,52 | 4,95 | 4,69 | 4,23 | 1,12 | 1,11 | -19,63 |
| control | 47 | 1,78 | 63,19 | 19,27 | 43,92 | 30,42 | -19,65 | -21,65 | -14,95 | -18,85 | 5,07 | 5,39 | 5,35 | 4,89 | 0,94 | 1,10 | -25,55 |
| control | 41 | 1,94 | 96,77 | 35,65 | 61,12 | 36,88 | -18,45 | -18,70 | -19,75 | -17,00 | 5,39 | 5,19 | 5,04 | 4,00 | 1,04 | 1,26 | -23,45 |
| control | 53 | 1,69 | 69,46 | 17,20 | 52,26 | 37,48 | -18,25 | -22,70 | -17,10 | -20,25 | 6,79 | 5,32 | 4,91 | 5,30 | 1,28 | 0,93 | -23,58 |
| control | 48 | 1,93 | 58,91 | 18,26 | 40,65 | 27,05 | -19,25 | -22,10 | -18,95 | -19,50 | 6,35 | 5,51 | 5,94 | 4,27 | 1,15 | 1,39 | -22,34 |
| control | 58 | 1,90 | 74,53 | 26,87 | 47,66 | 38,17 | -12,00 | -16,30 | -17,00 | -18,05 | 5,50 | 6,36 | 6,80 | 5,28 | 0,86 | 1,29 | -19,66 |
| control | 44 | 1,73 | 87,24 | 27,43 | 59,81 | 44,05 | -18,92 | -17,64 | -10,40 | -15,40 | 6,32 | 6,53 | 5,69 | 4,27 | 0,97 | 1,33 | -22,68 |
| control | 57 | 1,75 | 86,64 | 28,26 | 58,38 | 51,52 | -16,24 | -18,57 | -15,60 | -19,90 | 6,64 | 6,11 | 6,81 | 5,70 | 1,09 | 1,20 | -21,50 |
| control | 25 | 1,65 | 80,07 | 20,54 | 53,97 | 43,82 | -13,62 | -16,76 | -5,45 | -7,95 | 6,07 | 6,78 | 5,43 | 5,41 | 0,90 | 1,00 | -17,16 |
| control | 46 | 1,92 | 75,90 | 25,05 | 50,90 | 38,27 | -17,94 | -21,19 | -10,60 | -16,45 | 6,47 | 6,49 | 5,91 | 4,56 | 1,00 | 1,30 | -21,37 |
| control | 29 | 1,76 | 75,22 | 24,22 | 50,83 | 38,25 | -9,90 | -13,49 | -18,10 | -20,35 | 5,26 | 5,25 | 5,23 | 4,64 | 1,00 | 1,13 | -19,70 |
| MYBPC3 | 41 | 1,61 | 78,87 | 34,98 | 43,89 | 29,29 | -16,95 | -19,00 | -18,05 | -22,40 | 4,36 | 4,61 | 3,60 | 3,55 | 0,95 | 1,01 | -20,76 |
| MYBPC3 | 30 | 1,86 | 87,72 | 27,20 | 60,52 | 34,47 | -19,25 | -19,45 | -15,95 | -15,85 | 5,16 | 4,92 | 4,55 | 3,99 | 1,05 | 1,14 | -23,06 |
| MYBPC3 | 28 | 1,77 | 86,06 | 36,07 | 49,99 | 32,10 | -15,30 | -17,25 | -16,50 | -19,95 | 5,04 | 5,10 | 5,14 | 4,06 | 0,99 | 1,27 | -20,86 |
| MYBPC3 | 26 | 1,60 | 82,87 | 27,17 | 55,70 | 43,59 | -13,75 | -14,90 | -14,05 | -21,20 | 5,90 | 5,80 | 5,41 | 3,97 | 1,02 | 1,36 | -19,13 |
| MYBPC3 | 31 | 1,63 | 89,31 | 29,13 | 60,18 | 38,26 | -16,95 | -19,65 | -14,90 | -20,05 | 5,27 | 5,44 | 4,79 | 4,01 | 0,97 | 1,19 | -21,99 |
| MYBPC3 | 43 | 1,82 | 81,47 | 25,89 | 55,58 | 36,42 | -16,65 | -18,80 | -15,75 | -20,55 | 5,47 | 5,39 | 4,84 | 4,89 | 1,01 | 0,99 | -25,10 |
| MYBPC3 | 18 | 1,64 | 74,87 | 33,92 | 40,94 | 45,91 | -12,95 | -17,60 | -15,05 | -16,50 | 6,03 | 6,71 | 6,21 | 4,77 | 0,90 | 1,30 | -19,22 |
| MYBPC3 | 56 | 1,60 | 92,85 | 18,06 | 74,79 | 38,34 | -17,75 | -22,75 | -16,10 | -24,30 | 5,47 | 4,42 | 5,42 | 4,29 | 1,24 | 1,26 | -22,97 |
| MYBPC3 | 34 | 1,74 | 86,56 | 31,83 | 54,74 | 47,16 | -14,70 | -16,90 | -16,75 | -20,95 | 6,14 | 5,72 | 6,69 | 4,98 | 1,07 | 1,34 | -21,06 |
| MYBPC3 | 60 | 1,80 | 90,68 | 25,09 | 65,59 | 44,59 | -8,00 | -12,85 | -15,75 | -20,70 | 8,46 | 5,50 | 6,73 | 4,69 | 1,54 | 1,44 | -23,50 |
| MYBPC3 | 24 | 1,65 | 82,46 | 34,09 | 48,37 | 30,29 | -14,95 | -19,80 | -19,60 | -14,65 | 5,02 | 4,91 | 4,65 | 3,52 | 1,02 | 1,32 | -21,09 |
| MYBPC3 | 31 | 1,87 | 86,74 | 27,44 | 59,30 | 34,13 | -18,00 | -17,45 | -17,70 | -22,05 | 5,30 | 5,45 | 4,21 | 4,04 | 0,97 | 1,04 | -21,19 |
| MYBPC3 | 60 | 1,87 | 65,81 | 18,69 | 47,12 | 26,54 | -17,55 | -15,85 | -16,80 | -15,05 | 6,08 | 4,33 | 4,61 | 3,37 | 1,41 | 1,37 | -19,80 |
| MYH7 | 35 | 1,94 | 75,21 | 30,12 | 45,10 | 35,74 | -15,85 | -18,20 | -18,55 | -22,00 | 5,81 | 5,46 | 6,77 | 5,09 | 1,06 | 1,33 | -21,51 |
| MYH7 | 56 | 1,58 | 84,59 | 25,83 | 58,76 | 34,64 | -20,00 | -11,55 | -15,15 | -16,85 | 4,92 | 4,55 | 4,32 | 3,70 | 1,08 | 1,17 | -21,51 |
| MYH7 | 35 | 1,78 | 97,94 | 32,85 | 65,09 | 35,79 | -20,80 | -11,45 | -19,55 | -21,25 | 4,59 | 5,78 | 4,48 | 3,62 | 0,79 | 1,24 | -21,75 |
| MYH7 | 29 | 1,91 | 69,30 | 20,29 | 49,02 | 31,92 | -19,65 | -15,95 | -20,30 | -16,80 | 4,40 | 5,04 | 4,91 | 4,29 | 0,87 | 1,14 | -21,90 |
| MYH7 | 42 | 1,97 | 72,89 | 22,10 | 50,79 | 30,73 | -20,40 | -17,00 | -18,10 | -17,85 | 4,41 | 4,96 | 5,54 | 3,91 | 0,89 | 1,42 | -24,14 |
| MYH7 | 34 | 1,74 | 81,42 | 30,23 | 51,18 | 37,27 | -17,45 | -21,30 | -16,80 | -20,85 | 6,58 | 5,28 | 5,21 | 4,00 | 1,25 | 1,30 | -20,92 |
| MYH7 | 35 | 1,49 | 72,78 | 28,37 | 44,41 | 29,01 | -16,40 | -16,75 | -17,80 | -17,65 | 3,51 | 4,39 | 3,95 | 4,05 | 0,80 | 0,98 | -23,79 |
| MYH7 | 21 | 2,00 | 88,36 | 21,11 | 67,24 | 42,77 | -20,40 | -22,25 | -15,45 | -22,90 | 5,51 | 6,24 | 6,14 | 4,47 | 0,88 | 1,37 | -23,50 |
| MYH7 | 65 | 1,76 | 69,11 | 21,49 | 47,62 | 36,80 | -14,05 | -16,25 | -15,25 | -13,75 | 6,39 | 5,92 | 6,23 | 5,37 | 1,08 | 1,16 | -21,55 |
| MYH7 | 45 | 1,59 | 84,43 | 29,13 | 55,31 | 36,13 | -22,75 | -20,60 | -21,25 | -21,10 | 5,25 | 5,31 | 4,52 | 4,09 | 0,99 | 1,11 | -26,28 |
| MYH7 | 18 | 1,81 | 79,33 | 27,16 | 52,17 | 41,56 | -19,15 | -17,75 | -17,05 | -21,30 | 5,52 | 6,42 | 6,41 | 4,81 | 0,86 | 1,33 | -25,86 |
| TNNT2 | 58 | 1,70 | 54,73 | 18,10 | 36,63 | 31,49 | -12,75 | -14,85 | -13,05 | -21,05 | 4,71 | 5,74 | 7,29 | 4,56 | 0,82 | 1,60 | -16,36 |
| TNNT2 | 48 | 1,75 | 60,49 | 18,61 | 41,87 | 31,17 | -17,30 | -18,00 | -18,70 | -22,35 | 4,81 | 5,07 | 5,16 | 3,95 | 0,95 | 1,31 | -24,36 |
| TNNT2 | 59 | 1,77 | 65,52 | 18,14 | 47,37 | 36,70 | -17,95 | -23,15 | -13,90 | -24,35 | 4,81 | 7,70 | 5,41 | 3,94 | 0,63 | 1,37 | -22,03 |
| TNNT2 | 40 | 1,75 | 98,30 | 29,82 | 68,47 | 37,96 | -13,75 | -16,20 | -18,00 | -19,65 | 5,37 | 4,86 | 5,03 | 4,18 | 1,10 | 1,20 | -21,00 |
| TNNT2 | 26 | 1,53 | 74,75 | 18,49 | 56,26 | 27,72 | -17,60 | -22,40 | -18,00 | -25,55 | 3,88 | 4,23 | 3,58 | 3,12 | 0,92 | 1,15 | -24,04 |
| TNNT2 | 24 | 2,11 | 63,83 | 15,51 | 48,32 | 34,54 | -18,75 | -20,00 | -21,80 | -22,55 | 7,62 | 5,61 | 6,61 | 4,70 | 1,36 | 1,41 | -24,03 |

**Derived datasheet**
